# Apoptosis Induction via ATM Phosphorylation, Cell Cycle Arrest, and ER Stress by Goniothalamin and Chemodrugs Combined Effects on Breast Cancer-Derived MDA-MB-231 Cells

**DOI:** 10.1155/2018/7049053

**Published:** 2018-11-26

**Authors:** Patompong Khaw-on, Wilart Pompimon, Ratana Banjerdpongchai

**Affiliations:** ^1^Department of Biochemistry, Faculty of Medicine, Chiang Mai University, Chiang Mai 50200, Thailand; ^2^Laboratory of Natural Products, Faculty of Science, Lampang Rajabhat University, Lampang 52100, Thailand

## Abstract

Goniothalamin (GTN), a styryl-lactone, exhibits inhibitory effects on many kinds of cancer cells* in vitro*. The objectives of this study were to investigate the anticancer activities of GTN and molecular signaling pathways associated with cell death in human breast cancer MDA-MB-231 cell line. GTN inhibited the growth of MDA-MB-231 cells. Apoptosis was confirmed by annexin V-FITC and PI staining, and apoptotic morphology was observed by microscopy. Reduction of mitochondrial transmembrane potential and enhanced caspases activities were found in GTN-treated MDA-MB-231 cells. GTN significantly altered apoptosis-related protein expressions, including Noxa, PUMA, Bax, Bim, Bad, Bcl-2, Bcl-xL, and DIABLO, which was related to the gene expression levels. Mitochondrial calcium released to the cytosol and ER stress related proteins increased, which correlated with increases in ER stress gene expression levels. GTN induced hydrogen peroxide and superoxide anion radicals in MDA-MB-231 cells associated with cell cycle arrest in G2/M phase, which was induced by phosphorylation and* ATM *gene expression. Moreover, GTN had synergistic effects when combined with cyclophosphamide, 5-fluorouracil, paclitaxel, and vinblastine, and additive effect with methotrexate through caspases enzyme-acceleration. In conclusion, goniothalamin-induced MDA-MB-231 cell apoptosis occurred via intrinsic and extrinsic pathways, along with ER stress. These pathways provide new targeted drug strategies for advancements in anticancer medicine.

## 1. Introduction

Goniothalamin (GTN) is isolated and characterized from* Goniothalamus griffithii *[1]. It has been widely used as a folk medicine in south-east Asia. Goniothalamin exhibits an inhibitory effect on growth and the proliferation on various types of cancerous cell lines and noncancerous murine fibroblast (NIH3T3) cell line that were cultured* in vitro* [2]. GTN induces apoptosis in Jurkat T-cells via caspase-3,-7 and PARP-cleavage [3] and intrinsic apoptosis in human promyelocytic leukemia HL-60 cells [4]. Notably, the apoptosis induction pathway depends on the cancer cell type [1]. GTN induces cell cycle arrest at the G_2_/M phase in human breast cancer MDA-MB-231 cells [5]. In addition, GTN causes DNA damage, which subsequently leads to apoptosis in many cell lines [6–8].

Apoptosis, a programmed cell death, consists of death receptor- and/or mitochondria-mediated pathways. Chemical compounds, drugs, and ultraviolet (UV) light induce the mitochondrial pathway by generating mitochondrial stress with reduced mitochondrial transmembrane potential (MTP). After the death receptors bind with its ligands, the death receptor pathway initiates with Death-Inducing Signaling Complex (DISC) formation and triggers initiator caspase-8 followed by effector caspase-3 activation to induce cell death. Caspase-8 also cleaves proapoptosis Bid to become truncated Bid (tBid), which induces mitochondrial pore formation by Bax-Bax, Bax-Bak, or Bak-Bak dimers for the channel formation. Notably, pro- and antiapoptotic proteins have important roles in apoptosis pathways [9].

Reactive oxygen species (ROS) play a crucial role in apoptosis in cancer cells [10] since GTN causes oxidative damage in many types of cancer cells [11, 12]. ROS induces cancer cells to undergo apoptosis via interrupting the mitochondria oxidative phosphorylation, lipid peroxidation, and a double-strand DNA break [8]. The DNA break can induce cell cycle arrest by ATM/ATR activation, which is induced by p53 [13]. Furthermore, the p53-independent pathway has also been shown to be influential in the activation of DNA damage sensing molecules and proapoptosis proteins; PUMA, Noxa for apoptosis execution [14]. ER stress related proteins and heat shock 70 kD protein 5/GRP78/HSPA5 equilibrate cytosolic calcium, which is released from cellular organelle-induced apoptosis [15]. Ca^2+^ is released to the cytosol, while chaperone proteins and ER stress related proteins play crucial roles in programmed cell death induction in cancer cells [16].

Chemotherapeutic drugs have been developed and used for cancer treatments but are still associated with poor outcomes perhaps due to a lack of compliance and their complicated side effects. The use of combination treatments with rationale on different mechanisms also synergizes the effects of single targets and kills the cancer cells more effectively. Providing selective synergism against multiple targets, drug combinations are widely used and have become the leading choice for the treatment of cancer [17]. Targeted drug therapies are aimed directly at the cancerous cells or at the molecules that regulate or control the proliferation of cancer cells. These drugs are used in combination with other therapies for the advantages of fewer or less severe side effects [18]. Triple negative breast cancer MDA-MB-231 cells, which are characterized as negative for estrogen receptor (ER), progesterone (PR), and epidermal growth factor receptor (EGFR or HER2), become indicated of invasive breast cancer as a consequence of poor prognosis and have a potential to become drug-resistant [19]. In this study, the human invasive breast cancer MDA-MB-231 cell line was used as a model for an investigation whether GTN induced apoptosis, and its cytotoxic effects were considered when the treatment is combined with conventional chemodrugs, in addition to its related mechanism(s).

## 2. Materials and Methods

### 2.1. Chemicals

The leaves and twigs of* Goniothalamus griffithii *were collected in January 2011 from Chiang Mai Province, Thailand, and identified by the Forest Herbarium, Department of National Park, Wildlife and Plant Conservation, Ministry of Natural Resources and Environment, Bangkok, Thailand, where a voucher specimen (BKF16447) has been deposited. GTN was extracted, purified, and identified by Professor Wilart Pompimon, Lampang Rajabpat University, Lampang Province, Thailand. The phytochemical compound, goniothalamin, was isolated from* G. griffithii *as has been previously reported [20].

GTN stock solution was prepared in DMSO at 20 mM and GTN was diluted in Dulbecco's Modified Eagle Medium (DMEM, Gibco, Carlsbad, CA, USA). Annexin V Fluos-staining kit and Protease Inhibitor Cocktail tablets were obtained from Roche Diagnostics (Mannheim, Germany). Ficoll-Hypaque reagent (HISTOPAQUE®-1077), dihydroethidium (DHE) and 2′,7′–dichlorodihydrofluorescein diacetate (DCFH-DA), 3,3′-dihexyloxacarbocyanine iodide (DiOC_6_), and 3-(4,5 dimethylthiazol-2yl)-2,5 diphenyltetrazolium bromide (MTT) were obtained from Sigma-Aldrich, MO, USA. Rhod-2 acetoxymethyl (AM) ester and Fluo-3 AM were obtained from Thermo Fisher Scientific Inc., USA. Caspases-3, -8, and -9 determination kits were obtained from Invitrogen, Thermo Fisher Scientific Inc., USA. Primary and secondary antibodies and horse-radish peroxidase-conjugated antibodies were obtained from Abcam, Cambridge, UK. RNA isolation kit was obtained from GE Healthcare, UK. Total RNA was reversed to complementary DNA (cDNA) by using a cDNA Synthesis Kit and quantitative real-time PCR assays were performed with Reagents Kit (Bioline Reagents Ltd., USA).

### 2.2. Cell Culture

Triple negative breast cancer MDA-MB-231 cell line was cultured in DMEM, supplemented with 10% fetal bovine serum, penicillin, and streptomycin at 37°C in a humidified atmosphere of 5% CO_2_ at 37°C. After the cells grew to 80% confluence, the cell lines were trypsinized by 0.05% trypsin. Peripheral blood mononuclear cells (PBMCs) were isolated from heparinized blood obtained from adult volunteers as blood donors at Blood Bank Unit, Maha Raj Nakorn Chiang Mai Hospital, affiliated with the Faculty of Medicine, Chiang Mai University. Each blood donor was informed of the objectives and signed the written consent form in order to comply as individual volunteers, according to the Institutional Review Board, Research Ethics Committee, Faculty of Medicine, Chiang Mai University. PBMCs were separated from the whole blood of healthy volunteers by using Ficoll-Hypaque reagent as has been previously described [21].

### 2.3. Cell Viability Assay

The MTT (3-(4,5 dimethylthiazol-2yl)-2,5 diphenyltetrazolium bromide) assay [22] was performed by seeding MDA-MB-231 cells in 96-well culture plates. GTN treatment conditions were prepared in various concentrations and incubated for 24 hours. The cell viable activity in each well will be determined by MTT assay and compared to the untreated cells [23].

### 2.4. Drug Combination Assay

A process for analyzing the efficiency of combined drugs and the net effect was employed by the method of Chou TC and Talalay P [24]. Briefly, MDA-MB-231 cells were cotreated with various concentrations of GTN and conventional therapeutic drugs with a nonconstant ratio combination. The chemotherapeutic drugs that were used in this study include vinblastine, paclitaxel, 5-fluorouracil, methotrexate, and cyclophosphamide. After 24 hours of treatment, the cells were evaluated for viability using MTT assay and a function of the effect level (F_a_) values was calculated. The CompuSyn Software was employed for determination of combination index (CI) values and validated as a combination effect.

### 2.5. Fluorescence Microscopy

MDA-MB-231 cells were seeded on culture slides and treated with GTN. After 24 hours, the slides were fixed in ice-cold absolute alcohol and stained with 10 *μ*g/ml propidium iodide (PI). The slides were investigated for apoptotic morphology using a fluorescence microscope. Apoptosis positive cells were scored from 200 cells per trials of three independent experiments.

### 2.6. Apoptosis Assay

After treatment with GTN for 24 hours, the cells were trypsinized and the cell pellets were washed with phosphate-buffered saline (PBS). After that, the cells were stained with the reagent annexin V-fluorescein isothiocyanate (FITC) and PI for 15 minutes and were then processed using a flow cytometer.

### 2.7. Cell Cycle Analysis

Cell cycle analysis was performed on the goniothalamin-treated cells by using FlowCellect™ Cell Cycle Checkpoint ATM DNA Damage Kit (Merck Millipore Corporation, Germany) and operated by Guava EasyCyte 5HT Benchtop Flow Cytometer (Merck Millipore Corporation, Germany). The data were analyzed for p-ATM positive cells and the percentages of each phase in the cell cycle were recorded.

### 2.8. Assessment of Mitochondrial Transmembrane Potential / Cytosolic vs. Mitochondrial Calcium Ion Levels

MDA-MB-231 cells were treated with GTN for 24 hours. The cells were then combined with 40 nM 3,3′-dihexyloxacarbocyanine iodide (DiOC_6_); 250 nM Rhod-2 AM; and 10 *μ*M and Fluo-3 AM solutions. They were then incubated at 37°C for 15 minutes. The cells were washed twice with PBS and analyzed using the flow cytometry technique.

### 2.9. Intracellular Reactive Oxygen Species (ROS) Generation Assay

Intracellular ROS including superoxide anion radicals and hydrogen peroxide species were detected by fluorescence probes, viz., dihydroethidium (DHE) and 2′,7′-dichlorodihydrofluorescein diacetate (DCFH-DA), respectively, and then measured using a fluorescence microplate reader (BioTek, Winooski, VT, USA) [25, 26]. Briefly, MDA-MB-231 cells were preincubated with each probe for an hour and then treated with GTN at IC_20_ and IC_50_ concentrations for four hours with or without* N*-acetylcysteine to counter treatment of the ROS inhibitory effect during each oxidant production cycle.

### 2.10. Determination of Caspases-3, -8, and -9 Activities

Determination of caspases activity was performed by colorimetric protease assay. Briefly, the cell pellets were lysed with lysis buffer on ice and an equal amount of protein was prepared. Caspase-8 (IETD-*p*NA), caspase-3 (DEVD-*p*NA), and caspase-9 (LEHD-*p*NA) chromogenic substrates were added for an hour at 37°C. The optical density was measured at a wavelength of 405 nm using a microplate reader (BioTek, Winooski, VT, USA).

### 2.11. Immunoblotting

Proteins were extracted in RIPA containing supplements with Protease Inhibitor Cocktail tablets. The immunoblotting was performed as has been previously described [27]. Antibodies against Noxa, PUMA, Bax, Bim, Bad, p112-Bad, Bcl-2, Bcl-xL, DIABLO, GRP78, GADD153, Calreticulin, COX-4, and *β*-Actin were purchased from Abcam, UK.

### 2.12. Quantitative Real-Time Reverse Transcription-Polymerase Chain Reaction (Real-Time RT-PCR)

RNA was isolated from the cell pellets by using the Illustra RNAspin Mini Kit (GE Healthcare, UK). Total RNA was reversed to complementary DNA (cDNA) by using Tetro cDNA Synthesis Kit (Bioline Reagents Ltd., USA). Quantitative real-time PCR assays were performed with the SensiFAST™ SYBR® Lo-ROX Kit (Bioline Reagents Ltd., USA) and the QuantStudio™ 6 Flex Real-Time PCR System (Thermo Fisher Scientific Inc., USA). All the data were normalized by the* GAPDH* gene. The details of all gene primers are listed in Table 1.

### 2.13. Statistical Analysis

Data are presented as the mean ± standard deviation (S.D.) from triplicate trials of three independent experiments. The data were analyzed by one-way analysis of variance (ANOVA) with a comparison between groups of data by Tukey's test. All analyses were conducted using free statistic PSPP Software. Statistical significance was considered when *∗ p *< 0.05 or *∗∗ p* < 0.01.

## 3. Results

### 3.1. Cytotoxic Effect and Cell Cycle Arrest on MDA-MB-231 Cells after Treatment with Goniothalamin

**Figure 1:**
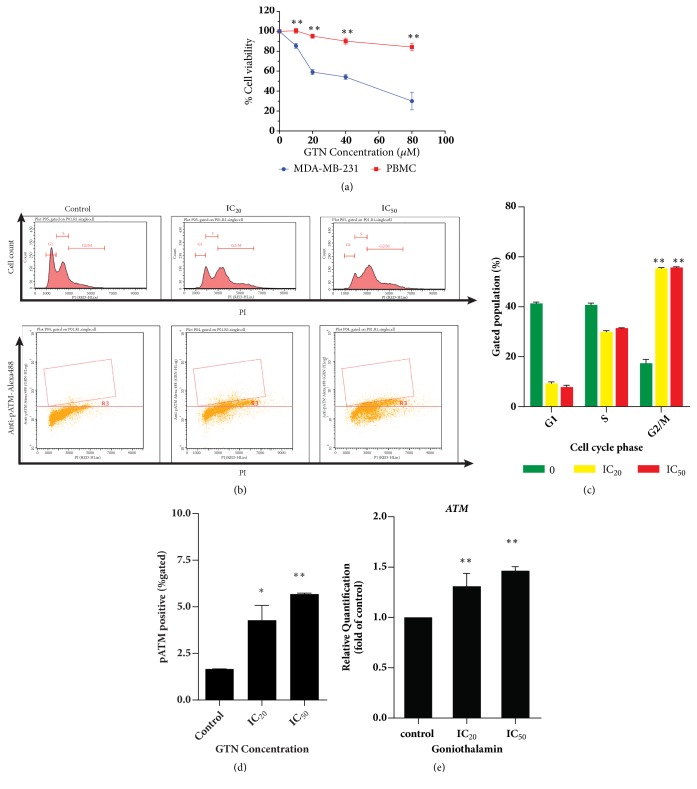
Cytotoxicity of goniothalamin (GTN) against human breast cancer MDA-MB-231 cells. Percent cell viability (a) of adherent MDA-MB-231 compared with PBMCs as mean ± SD. Cell cycle analysis of MDA-MB-231 cells after treatment with GTN at IC_20_ and IC_50_ ((b) upper panel). Dot plot of cells positive for phospho-ATM was accomplished by using specific Guava® Cell Cycle reagent ((b), lower panel). Bar graphs of percent cells in each phase of the cell cycle are shown as mean ± SD analyzed by using Guava® Flow Cytometry easyCyte™ Systems (c). Bar graphs of cells that were positive for phospho-ATM as mean ± SD (d). Bar graphs are presented of* ATM* gene expression in GTN-treated MDA-MB-231 cells (e). The significance of statistical values compared to control (without treatment) was marked with *∗*,* p*<0.05; *∗∗*,* p*<0.01.

To determine the cytotoxic effect and cell cycle distribution histogram of goniothalamin-treated MDA-MB-231 cells, GTN was found to be toxic against MDA-MB-231 cells at 24 hours of treatment (Figure 1(a)) with an inhibitory concentration of 50 percent (IC_50_) at 37 *μ*M. However, GTN was found to be less toxic to PBMCs as a normal cell control with IC_50_ of > 80 *μ*M. Cell cycle progression is also an event responding to DNA damage, which is usually signaled via the ATM/ATR/p53/p21 pathway [28]. Cell cycle and phosphorylated ATM were investigated and analyzed by flow cytometry and are illustrated in Figure 1(b). Consequently, GTN-treated cells were arrested at G_2_/M phase (Figure 1(c)). Since p-ATM functions in DNA injuries or genome instability, it is a marker of cancer cell DNA damage [29]. Phosphorylated ATM increased significantly in a dose-dependent manner (Figure 1(d)). Moreover, the gene expression levels of* ATM *increased in GTN-treated MDA-MB-231 cells in a dose-dependent manner (Figure 1(e)).

### 3.2. Apoptosis Induction and the Pathways of GTN-treated MDA-MB-231 Cell Death

**Figure 2:**
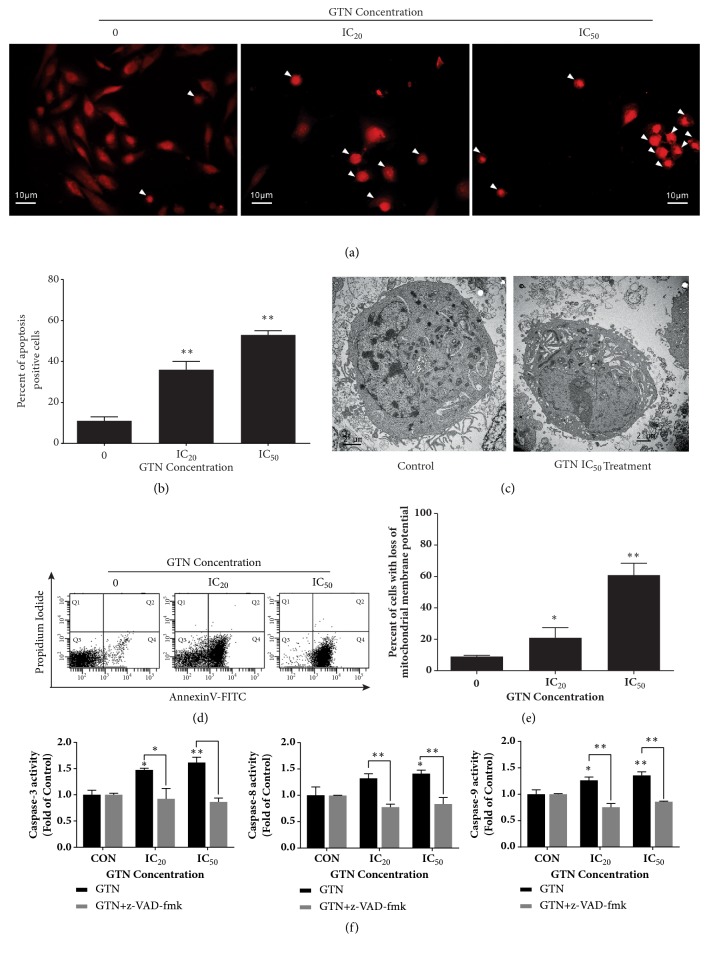
Apoptosis induction by GTN in MDA-MB-231 cells. Cell morphology of apoptotic cells was condensed nuclei and apoptotic bodies (arrows) after staining with propidium iodide (PI) (a). Bar graphs of percent apoptotic cells were obtained from counting positive apoptotic cells stained with PI of total 200 cells in a sample from three independent experiments as mean ± SD (b). A transmission electron micrograph of the representative apoptotic cell that was compared to the normal cell after GTN treatment for 24 h (c). Dot plots of early apoptosis were confirmed by staining with annexin V-FITC and PI employing flow cytometry (d). Bar graphs of early apoptotic cells as evidenced by positive cells for annexin V-FITC as mean ± SD (e). Caspases-3, -8 and -9 activities increased dose dependently after being incubated with GTN at IC_20_ and IC_50_ for 24 hours and they were then compared to control. The three caspase activities were attenuated by pre-treatment for an hour with z-VAD-fmk at the concentration of 10 *μ*M (f). The significance of statistical values compared to control (without treatment) was marked with *∗*,* p*<0.05; *∗∗*,* p*<0.01.

To investigate apoptosis in GTN-treated breast cancer MDA-MB-231 cells, fluorescence micrographs revealed the typical apoptosis morphology as condensed nuclei and apoptotic bodies (see Figure 2(a)). The apoptotic positive cells from fluorescence micrographs were scored, which increased in a dose-dependent manner (see Figure 2(b)) and transmission electron micrographs also exhibited apoptotic cell death (Figure 2(c)). GTN-treated cells also increased annexin-V-FITC signals dose-dependently, as early apoptosis characters (Figure 2(d)). To study the pathways of GTN-induced apoptosis, cancer cells exhibited the disruption of mitochondrial transmembrane potential (MTP) or loss of MTP (Figure 2(e)). Caspases-3, -8, and-9 activities also increased significantly in a dose-dependent manner and this effect was attenuated by a pan-caspase inhibitor (z-VAD-fmk) (Figure 2(f)).

### 3.3. Apoptosis-Related Proteins and Gene Expression Alterations in GTN-treated MDA-MB-231 Cells

**Figure 3:**
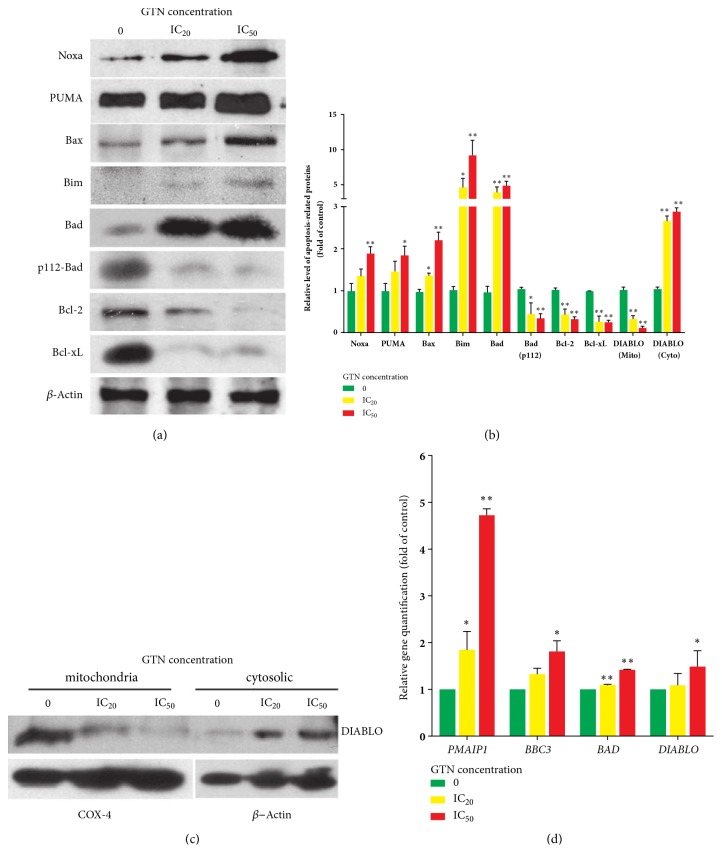
Alteration of apoptosis-related proteins and gene expressions in Bcl-2 family, pro-apoptotic, such as Bax and BH3-only proteins, e.g., Noxa, PUMA, Bim, Bad, phospho112-Bad, and anti-apoptotic proteins in Bcl-2 family such as Bcl-2, Bcl-xL were determined by Western blotting (a). The relative levels of protein expressions of MDA-MB-231 cells treated with GTN were obtained using densitometry from three independent experiments of Western blotting as mean ± SD (b). DIABLO, a protein in the intermembranous space of the mitochondria, was released into the cytosol in apoptotic cells. The amount of DIABLO protein increased in the cytoplasm and decreased in mitochondria detected by using digitonin-induced-cell fractionation followed by Western blotting (c). Some related corresponding gene expressions were measured by real-time RT-PCR and calculated using the ^∆∆^Ct method for relative quantifications. The data are shown as mean ± SD from three independent experiments (d). The significance of statistical values compared to the control (without treatment) was marked with *∗*,* p*<0.05; *∗∗*,* p*<0.01.

To investigate the roles of Bcl-2 family proteins in signaling pathways, GTN-treated MDA-MB-231 cells were investigated by Western blotting and band density analyses as shown in Figures 3(a) and 3(b). The proapoptosis proteins levels, Noxa, PUMA, Bax, Bim, and Bad, increased, whereas the antiapoptotic proteins Bcl-2 and Bcl-xL decreased. Bad protein was also activated by dephosphorylating at serine 112. Mitochondrial intermembranous space protein, DIABLO, was also released from mitochondria to the cytosol after GTN treatment in a dose-dependent manner, which is illustrated in Figures 3(a), 3(b), and 3(c). The expressions of apoptotic related-genes including* PMAIP1/Noxa*,* BBC3/PUMA*,* BAD,* and* DIABLO* also increased after MDA-MB 231 cells were treated with GTN (Figure 3(d)).

### 3.4. GTN-Activated Reactive Oxygen Species (ROS) Generation and Cellular Calcium Ion Levels Alteration-induced-ER Stress in MDA-MB-231 Cells

In the apoptosis of cancer cells, the ROS generation and ER stress occur as pathways of apoptotic cell death signaling [13]. Thus, to investigate whether or not GTN induced ROS production in MDA-MB-231 cells, intracellular ROS were determined by using DCFH-DA and DHE assays.

**Figure 4:**
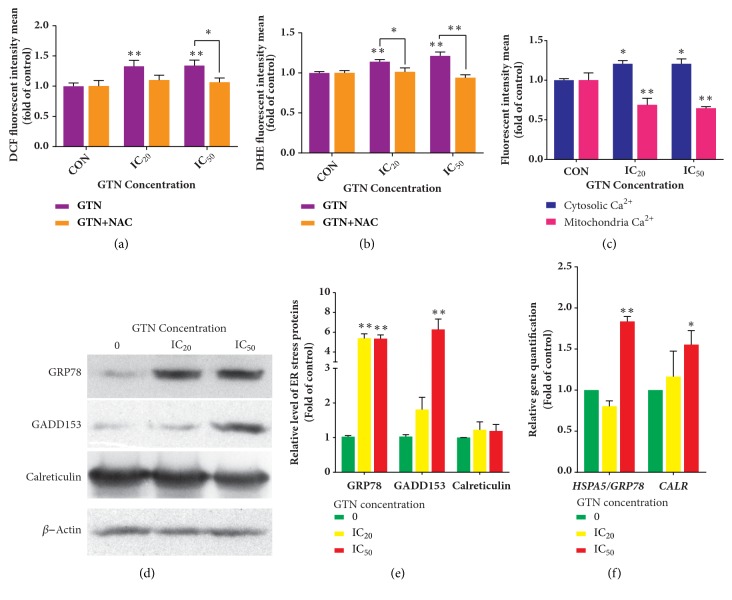
Reactive oxygen species (ROS) production and alteration of endoplasmic reticulum (ER) Calcium ion levels and ER protein expressions. ROS generation is exhibited as mean ± SD of fluorescence intensity in folds compared to that of the control (without treatment) from three independent experiments by using fluorescence probes: DCFH-DA (a) and DHE (b) measured by a fluorescence microplate reader.* N*-acetylcysteine (NAC) was used to confirm the oxidative stress involvement in the signaling pathway. ER stress was determined by Ca^2+^ ion level determination probes in the cytoplasm and mitochondria. ER stress was exhibited as evidenced by the increased levels of Ca^2+^ in the cytosol and decreased in the mitochondria. The data were shown as mean ± SD of fluorescence intensity as folds compared to the control (without treatment) from three independent experiments (c). The ER chaperone protein expression levels, GRP78, GADD153, and Calreticulin, were illustrated by Western blotting after MDA-MB-231 cells were treated with GTN at IC_20_ and IC_50_ for 24 hours (d). The densitometry of protein bands was calculated by Image J Software and is shown as mean ± SD of three independent experiments (e). Bar graphs of mRNA relative levels of* HSPA5/GRP78* and* CALR* by real-time RT-PCR and results were calculated by using the ^∆∆^Ct method for relative quantifications. The data are presented as mean ± SD from three independent experiments (f). The significance of statistical values compared to the control (without treatment) was marked with *∗*,* p*<0.05; *∗∗*,* p*<0.01.

The ROS levels in GTN-induced MDA-MB-231 cells were measured in time variations using GTN at 50 *μ*M. It was exhibited that the DCF fluorescence intensity levels significantly increased after an hour when compared to the control (Supplement Figure 1). However, the DCF intensity slightly increased at 1 to 12 hours of treatment and then displayed a trend to decrease to a normal level after 6 hours of treatment, but not significantly (*p* > 0.05) (as shown in Supplement Figure 1). Hence, we chose to investigate this result by employing concentration variations instead and used a constant GTN-incubation time at an hour. After GTN treatment, dichlorofluorescein (DCF) and ethidium (E) mean fluorescence intensities significantly increased in GTN-treated MDA-MB 231 cells, for which* N*-acetylcysteine (NAC), an antioxidant, attenuated the effect of GTN-mediated-intracellular ROS generations, both as hydrogen peroxide and superoxide anion radicals (Figures 4(a) and 4(b)), respectively.

To determine whether or not the cellular calcium induced ER stress, Fluo-3 AM fluorescence dye as a cytosolic calcium ion level marker was used and its intensity also increased in the GTN-treated MDA-MB-231 cells. However, Rhod-2 AM fluorescence dye, as a mitochondrial calcium ion marker, revealed that its fluorescence intensity decreased significantly in a dose-dependent manner, but the calcium ion concentrations were inversely related to each compartment, i.e., high in the cytosol and low in mitochondria (Figure 4(c)). In addition, ER stress* HSPA5/GRP78 *mRNA and HSPA5/GRP78 protein levels and GADD153 protein levels increased after GTN treatment in MDA-MB 231 cells. Another ER stress gene,* CALR*, increased significantly only in mRNA level, but the expression of Calreticulin protein level did not change (Figures 4(d), 4(e), and 4(f)).

### 3.5. Goniothalamin and Conventional Chemotherapeutic Drugs Combination Effect on MDA-MB-231 Cells

**Figure 5:**
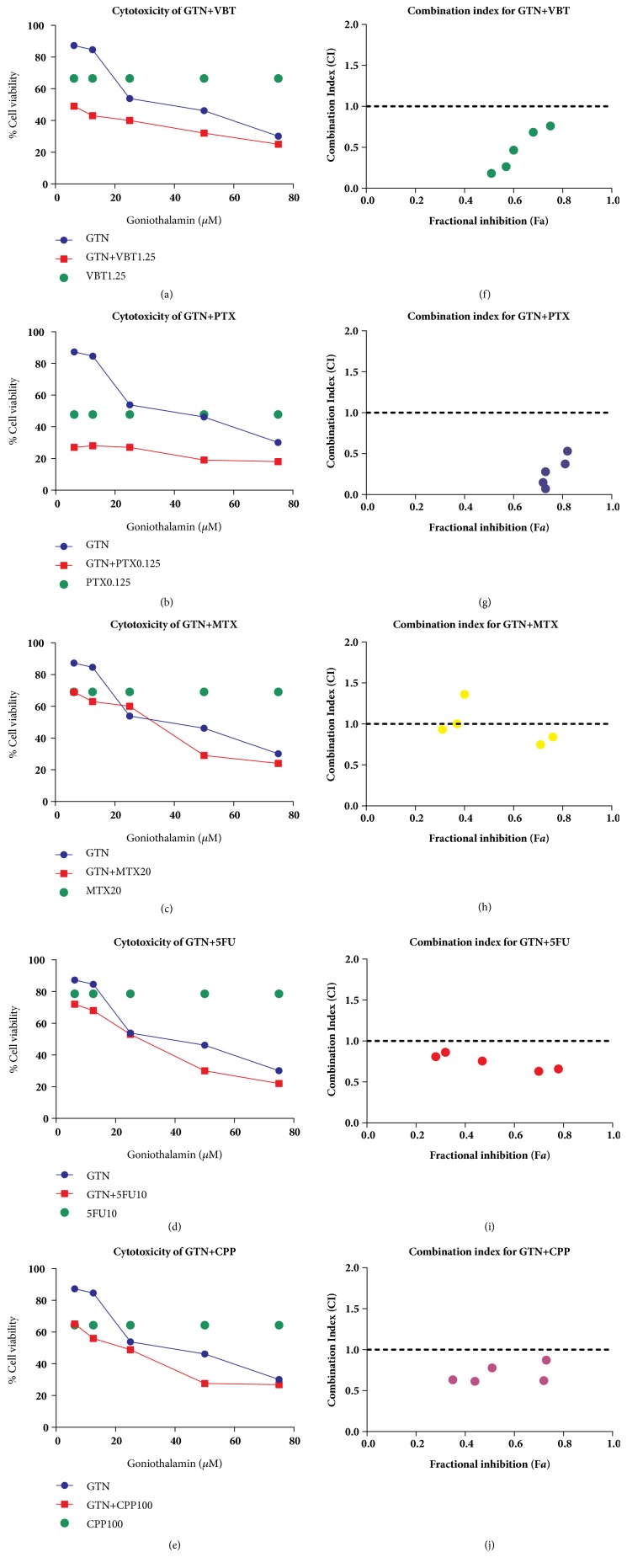
Combined effects of GTN and 5 chemodrugs on MDA-MB-231 cells. The non-constant ratio combination of cytotoxicity of 6.25, 12.5, 25, 50 and 75 *μ*M GTN and fixed concentration chemodrugs: 1.25 *μ*M vinblastine (VBT) (a), 0.125 *μ*M paclitaxel (PTX) (b), 20 *μ*M methotrexate (MTX) (c), 10 *μ*M 5-fluorouracil (5-FU) (d) and 20 *μ*M cyclophosphamide (CPP) (e). The cell viability was measured by MTT assay. Combination index (CI) plot corresponds to the cell viabilities of each drug; vinblastine (VBT) (f), paclitaxel (PTX) (g), methotrexate (MTX) (h), 5-fluorouracil (5-FU) (i) and cyclophosphamide (CPP) (j), which were interpreted from CompuSyn Software analysis.

**Figure 6:**
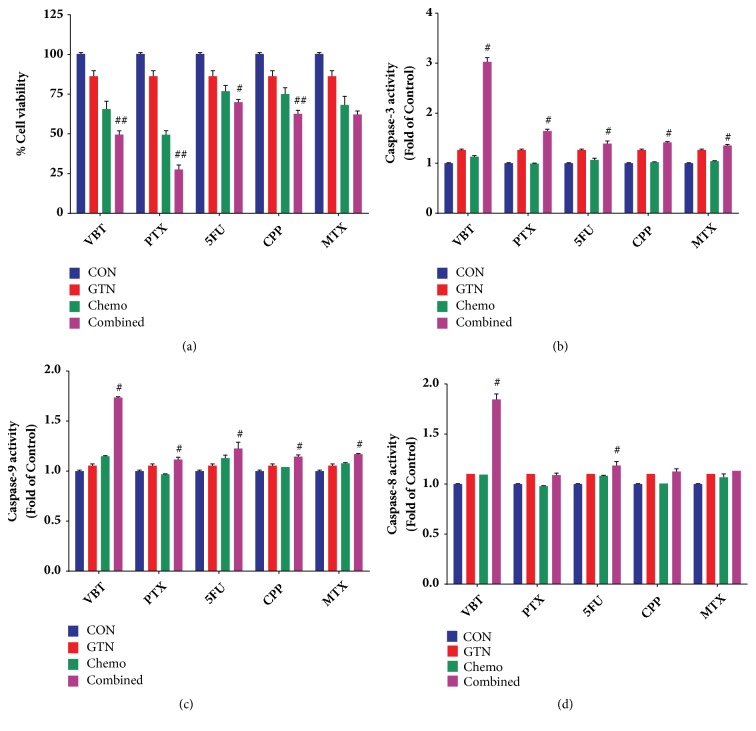
Combined effects of GTN and 5 chemodrugs on MDA-MB-231 cells. The cytotoxicity of 6.25 *μ*M GTN and chemodrugs: 0.125 *μ*M paclitaxel (PTX), 1.25 *μ*M vinblastine (VBT), 20 *μ*M methotrexate (MTX), 10 *μ*M 5-fluorouracil (5-FU) and 20 *μ*M cyclophosphamide (CPP); in non-constant ratio combination by fixed chemodrug concentrations and cell viability was measured by MTT assay (a). Caspase-3 (b), caspase-9 (c) and caspase-8 (d) activities were measured in GTN and chemodrug combinations were performed by using their specific substrates. The significance of statistical values compared to single treatments was marked with #,* p*<0.05 and ##,* p*<0.01.

A recent study has shown that the combination of a natural product and conventional chemodrugs is widely used for cancer treatment, which has been found to reduce side effects and increase therapeutic outcomes [30]. To conduct drug combination effects of GTN with chemotherapeutic drugs in MDA-MB-231 cells, the cytotoxic effect of the drug combinations was assessed in nonconstant ratio combinations by varying GTN concentrations and fixing the chemodrug concentration values to minimize the chemodrug adverse effects (see Figures 5(a)–5(e)). The cell viability of drug combinations was illustrated by their statistical significance when compared with single GTN or chemodrug treatments alone (see Figures 5(a), 5(c), 5(d), 5(e), and 6(a)). Combination index (CI) value was calculated by CompuSyn Software. The combination of 6.25 *μ*M GTN with 0.125 *μ*M paclitaxel (PTX), 1.25 *μ*M vinblastine (VBT), 10 *μ*M 5-fluorouracil (5-FU), or 100 *μ*M cyclophosphamide (CPP) revealed a synergistic effect for which CI was less than 1.0 and the additive effect in 20 *μ*M methotrexate (MTX) combination with CI value was estimated to equal 1.0 (see Table 2 and Figures 5(f)–5(j)). The synergistic effect of GTN was also exhibited in caspases-3 and -9 activities, which markedly increased when compared with GTN alone or in single chemodrug treatments, but caspase-8 activity significantly increased in GTN-combined treatments with VBT and 5-FU (see Figures 6(b)–6(d)).

## 4. Discussion

GTN was previously reported to possess anticancer properties against various cancer cells via intrinsic and extrinsic apoptotic pathways [31]. In this study, the cytotoxicity also exhibited the fifty-percent inhibition growth concentration at 37 *μ*M in the MDA-MB-231 cell line for 24 hours of incubation (Figure 1(a)). The general janitor genes are DNA repair and mitotic checkpoint genes. DNA damage checkpoint genes, ataxia telangiectasia mutated (*ATM*), and the tumor suppressor* p53* induce apoptosis in response to DNA damage [32]. Activation of ATM by autophosphorylation on Ser1981 occurs in response to exposed DNA double-stranded breaks. Several downstream targets have been identified. These substrates include many tumor suppressors, such as p53 which respond to the S-phase checkpoint and BRCA1 to facilitate the G_2_ checkpoint, respectively [33]. The triple negative breast cancer MDA-MB-231 cell line has a mutant tumor suppressor p53, but it has a wild type of BRCA1 that is responsible for DNA double-strand break and G_2_ cell cycle arrest [34]. GTN has been reported to induce DNA double-strand breaks in oral cancer cells which induced apoptosis [12].

The mode of MDA-MB-231 cell death was apoptosis confirmed by the morphologies under fluorescence and transmission electron micrographs (Figures 2(a) and 2(c)). The externalization of phosphatidylserine (PS) to the outer layer of the cell membrane also increased, suggesting early apoptosis in cell death mode. Percentage of cells in early apoptosis increased in a dose-dependent manner, which involved the cells in the right lower quadrant (annexin V-FITC-positive cells, PI-negative cells) (Figure 2(d)). Percentage of cells with a loss of mitochondrial transmembrane potential increased dose-dependently (Figure 2(e)), indicating the influence of the apoptosis and regulated necroptosis pathways. The caspases-3, -8, and -9 activities were activated when compared to the control (untreated cells). However, the pan-caspase inhibitor, z-VAD-fmk, attenuated all three caspases activities when compared to the GTN-treated cells individually (Figure 2(f)). Cumulatively, at this point, the apoptosis pathways of GTN-induced MDA-MB-231 cells were completed via both mitochondrial and death receptor (caspase-8) pathways.

Furthermore, Bcl-2 family proteins and genes play significant roles in apoptotic cell deaths [35]. Hence, GTN-induced apoptotic cells were investigated for the expressions of some of the Bcl-2 family proteins and corresponding mRNAs. The proapoptotic Bax, BH3-only; Noxa, PUMA, Bim, Bad, and p112-Bad; and antiapoptotic Bcl-2 and Bcl-xL proteins were measured quantitatively by Western blotting and densitometry. It exhibited that Bax and BH3-only proteins were upregulated, and antiapoptotic proteins were downregulated when compared to the control. This study also demonstrated the activation of Bad as dephosphorylated-Bad and the high expression of Bim (Figures 3(a) and 3(b)), which are responsible for DNA damage, cell cycle arrest, and ATM activation via ERK activation by GTN [11, 36]. The mitochondrial pathway was also established by the DIABLO release from mitochondrial intermembranous space. DIABLO, which is located in the mitochondrial intermembranous space, functions to inhibit a family of proteins called “inhibitors of apoptosis protein (IAP)”. Therefore, DIABLO plays a role in apoptosis induction. We illustrated that DIABLO levels in mitochondria were abrogated and in cytosol were enhanced in a concentration-dependent manner (Figures 3(b) and 3(c)). However, the levels of mRNA expression of some apoptotic genes, such as* PMAIP1/noxa, BBC3/PUMA, bad*, and* DIABLO*, were determined by real-time RT-PCR and compared with the protein levels by immunoblotting and densitometer. The mRNA expression levels were found to correspond to the protein expression levels.

Oxidative stress is reported to induce cancer cell death via ROS/RNS production in signaling via the mitochondrial pathway [21]. The roles of oxidative stress in GTN-induced apoptosis were investigated and reported as the major causes of apoptosis in many cancer cell types, namely, Jurkat T-cells, melanoma cells, and Ca9-22 oral cancer cells [8, 11, 12, 37]. This study focused on which types of ROS that GTN were induced by DCFH-DA and DHE relative fluorescence intensity, and this was confirmed by the reduction of fluorescence intensity when the cancer cells were pretreated with* N*-acetylcysteine (NAC) (Figures 4(a) and 4(b)), an antioxidant molecule. The reactive oxygen species including hydrogen peroxide was similarly reported in the previous investigation using DCFH-DA as a probe [31] and superoxide anion radicals, which was first shown in this study by employing DHE as an indicator [38]. The increase of ROS demonstrated by both DCF and E fluorescence intensity contained an increased trend of dose response manners. However, there are many factors that can influence the levels of ROS generation in the cells, such as the levels of reduced glutathione, NADPH reducing agent (a coenzyme), and the status of mitochondrial electron transport chain [39]. These might affect the levels of ROS production that are induced by GTN-treatment, which caused the response to not be determined in a concentration manner remarkedly, of both hydrogen peroxide and superoxide anion radicals.

Calcium ion levels also alter apoptosis and other kinds of cell death, such as necroptosis [21]. Hence, the calcium ion levels were measured using fluorescence dye Rhod-2 AM, specific for mitochondrial compartment, whereas Fluo-3 AM dye was used specifically for Ca^2+^ levels in the cytosolic compartment. Calcium ion levels in the cytoplasmic compartment were higher, while those in the mitochondria were lower in GTN-treated breast cancer MDA-MB-231 cells when compared to the control (untreated cells) (Figure 4(c)). Intracellular ROS and calcium ions levels were involved in oxidative and endoplasmic reticulum (ER) stresses-induced apoptosis [40]. ER stress is also considered as a mechanism of cancer cell apoptosis [41]. Consequently, the expressions of some ER stress proteins and mRNAs were investigated, such as GRP78 (glucose-regulated protein 78), GADD153 (growth arrest- and DNA damage-inducible gene 153) and Calreticulin proteins and* HSPA5/GRP78 *and* CALR* mRNA levels. The ER stress proteins, HSPA5/GRP78 and GADD153, were upregulated (as shown in Figures 4(d), 4(e), and 4(f)), except for Calreticulin which did not change. This might have been due to the transient mRNA expression of* CALR *mRNA, whereas the protein half-life is short and is degraded quickly [42, 43]. Calreticulin has many biological functions under both physiological and pathological conditions. Calreticulin is located at the cell membrane and functions as an “eat me” signal and brings the cancer cells to their microenvironment, such as by the extracellular matrix (ECM) in an inside-out or outside-in signaling manner through integrins or the cytoskeletal protein network. It also plays a role in angiogenesis, metastasis, cell adhesion, and tumorigenesis [44]. HSPA5/GRP78 functions as a chaperone in protein proper folding in ER [45]. Another ER stress-mediated apoptosis protein is the C/EBP homologous protein (CHOP), which is also known as growth arrest- and DNA damage-inducible gene 153 (GADD153), which downregulates Bcl-2 and perturbs cancer cell oxidation/reduction or the redox state [46].

Combination chemotherapy has been widely used in breast cancer treatments. Drug combinations can improve prognosis by overcoming drug resistance in invasive breast cancer patients [47]. Currently, natural products have become a potential strategy for drug combinations in cancer therapies. Phytochemical compounds are less toxic to normal cells and have a different target when compared to conventional chemotherapeutic drugs [48]. A synergistic effect is an enhanced effect when two or more compounds are combined, which improves toxicity while minimizing the adverse effects. Mostly, a nonconstant combination ratio is used to reduce the adverse effects of chemodrugs [49]. The cytotoxicity of commonly used chemodrugs on human breast cancer MDA-MB-231 cells has been previously reported [50]; the same individual concentration which was used in this study ranged from twenty to fifty percent inhibitory concentrations and displayed less toxicity than twenty percent in the normal cells (data not shown). The additive effect of GTN and MTX, a slightly synergistic effect for GTN and 5-FU or CPP, was grouped by drug actions as antimetabolites or alkylating agents, which were less sensitive to triple negative breast MDA-MB-231 cancer cells [51]. However, mitotic spindle targeted chemodrugs, PTX and VBT, had a synergistic effect when combined with GTN (Figure 5(a) and Table 2), because low concentrations of PTX and VBT increased arrest in mitosis [52]. The synergistic effect when combined with GTN is a target via mitotic arrest (Figure 1(c)). All combinations of GTN with chemodrugs enhanced the apoptotic caspases-3 and -9 activities, whereas those of caspase-8 increased in VBT and 5-FU plus with GTN (Figures 6(b)–6(d)). The mechanisms of drug combinations with GTN treatments showed synergistic effects via the mechanisms that are required to be illustrated, while the molecular signaling pathways require further study to clarify the apoptotic and/or mitotic arrest signaling pathways.

## 5. Conclusion

**Figure 7:**
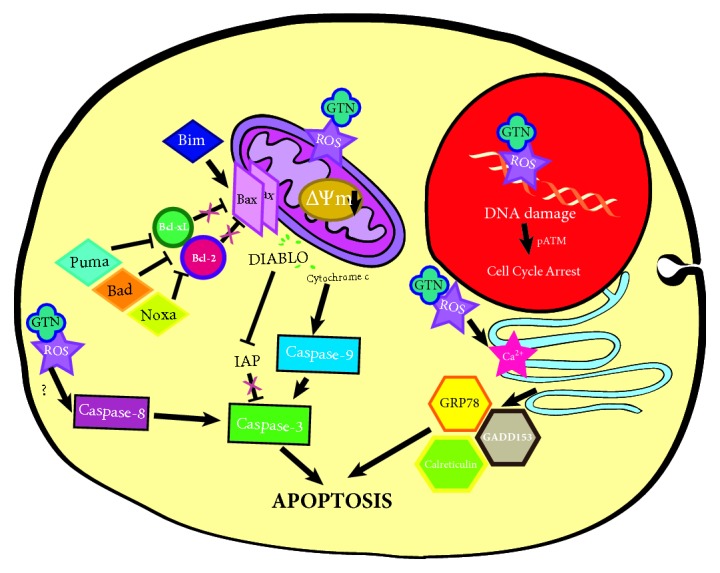
Illustration representing the mechanism of goniothalamin (GTN)-induced human breast-derived MDA-MB-231 cancer cell apoptosis. GTN induced apoptosis by DNA damage from oxidative stress and an increase of ROS production. GTN directly induced mitochondria-mediated stress leading to ROS production, mitochondrial intermembranous protein release and activation with sequential caspases. GTN induced a cellular stress response, which led to the upregulation of pro-apoptotic molecules. GTN also induced ER-stress and an increase in the levels of the ER-stress proteins leading to apoptosis.

Goniothalamin induced adherent human breast cancer MDA-MB-231 cells apoptosis via the intrinsic and extrinsic pathways initiated by intracellular oxidative and ER stresses, which altered Bcl-2 family proteins/mRNAs levels and ER chaperone proteins/mRNA expression levels. G2/M cell cycle arrest was illustrated in GTN-treated MDA-MB-231 cells. Notably, levels of p-ATM increased indicating DNA instability in breast cancer cells after GTN treatment. The novel findings of this study included the illustration of ER and oxidative stress pathways, phosphorylated-ATM, DNA damage, or instability and combined five chemotherapeutic drugs with GTN to demonstrate the relevant synergistic and additive effects. The synergistic combination effect of GTN was exhibited in cell viability reduction when combined with chemodrugs, which also enhanced some caspase activities as evidenced by the intrinsic and/or extrinsic apoptosis pathways. The signaling pathway of GTN-induced cell death is illustrated in Figure 7. GTN has a high potential as a breast cancer treatment agent and as a single natural compound or as one that could be used in combination with conventional chemotherapeutic drugs to reduce the required dosage of effective chemotherapeutic drugs and to cause less severe adverse side effects.

## Figures and Tables

**Table 1:** . Primers for specific genes in the real-time RT-PCR method.

**Symbol**	**Synonym**	**Name**		**Sequence of primers (5**′**->3**′**)**
***GAPDH***		glyceraldehyde-3-phosphate dehydrogenase	F	TGCACCACCAACTGCTTAGC
			R	GGCATGGACTGTGGTCATGAG
***BAD***		BCL2 associated agonist of cell death	F	GCACAGCAACGCAGATGC
			R	AAGTTCCGATCCCACCAGG
***DIABLO***	*smac*	diablo IAP-binding mitochondrial protein	F	GAAGCTGGAAACCACTTGGATGA
			R	TGAATGTGATTCCTGGCGGTTA
***BBC3***	*PUMA*	BCL2 binding component 3	F	GCAGGCACCTAATTGGGCT
			R	ATCATGGGACTCCTGCCCTTA
***PMAIP1***	*NOXA*	phorbol-12-myristate-13-acetate-induced protein 1	F	GCTGGAAGTCGAGTGTGCTA
			R	CCTGAGCAGAAGAGTTTGGA
***HSPA5***	*GRP78*	heat shock protein family A (Hsp70) member 5	F	GCCTGTATTTCTAGACCTGCC
			R	TTCATCTTGCCAGCCAGTTG
***CALR***		calreticulin	F	AAATGAGAAGAGCCCCGTTCTTCCT
			R	AAGCCACAGGCCTGAGATTTCATCTG
***ATM***		ATM serine/threonine kinase	F	TGCCAGACAGCCGTGACTTAC
			R	ACCTCCACCTGCTCATACACAAG

**Table 2:** GTN and chemodrug concentrations in combination treatments and the combination index (CI) used to determine the synergistic combination effects calculated using CompuSyn Software.

	**Goniothalamin (GTN) (** ***μ*** **M)**					**Combination Index (CI)**
	6.25	6.25	6.25	6.25	6.25	
**Paclitaxel (PTX), (** ***μ*** **M)**	0.125					0.0709

**Vinblastine (VBT), (** ***μ*** **M)**		1.25				0.18271

**5-Fluorouracil (5FU), (** ***μ*** **M)**			10			0.80856

**Cyclophosphamide (CPP), (** ***μ*** **M)**				100		0.63287

**Methotrexate (MTX), (** ***μ*** **M)**					20	0.93235

## Data Availability

The data collected in the present study are properly analyzed and summarized in Methods and Results, and all are available from the corresponding author upon reasonable request. All materials used in this study are properly included in Methods.

## Supplementary Materials

Supplementary Materials
** Supplement Figure** **1** A Bar graph demonstrating DCF intensity of 50 *μ*M GTN treatment in MDA-MB-231 cells for 1* to *24 hours. It exhibited significant alteration at* an* h*our* when compared to without treatment* (p < 0.05) *but* there* was no significant change among groups at 1* to *12 h*ours* (*p* > 0.05).
