# Supplementary material for: Apoptosis Induction via ATM Phosphorylation, Cell Cycle Arrest, and ER Stress by Goniothalamin and Chemodrugs Combined Effects on Breast Cancer-Derived MDA-MB-231 Cells
Source: Biomed Res Int. 2018 Nov 26;2018:7049053. doi: 10.1155/2018/7049053 (PMC6287143; doi:10.1155/2018/7049053)
Supplement: Supplementary Materials — Supplement Figure 1 A Bar graph demonstrating DCF intensity of 50 μM GTN treatment in MDA-MB-231 cells for 1 to 24 hours. It exhibited significant alteration at an hour when compared to without treatment (p < 0.05) but there was no significant change among groups at 1 to 12 hours (p > 0.05). [file 7049053.f1.pdf]

Supplementary Figure 1

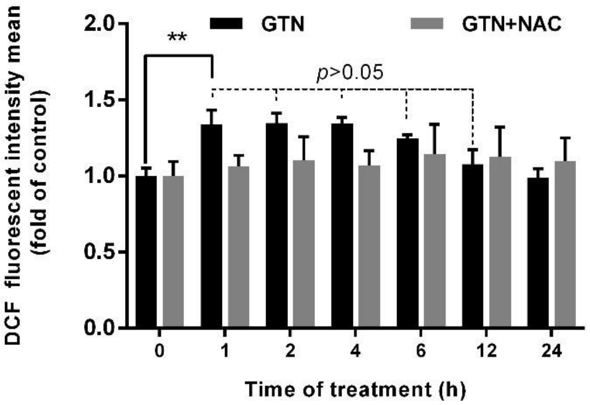

Supplement Figure 1 A Bar graph show*ed* DCF intensity of 50 µM GTN treatment in MDA-MB-231 cells for 1 *to* 24 hours. It exhibited significant alteration at *an* h*our* when compared to without treatment *(*$p < 0.05$*)* but *there* was no significant change among groups at 1 *to* 12 h*ours* ($p > 0.05$)
